# The complex case of *Macaronichnus* trace fossil affecting rock porosity

**DOI:** 10.1038/s41598-021-81687-6

**Published:** 2021-01-21

**Authors:** Javier Dorador, Francisco J. Rodríguez-Tovar, Olmo Miguez-Salas

**Affiliations:** 1 Department of Earth Sciences, Royal Holloway University of London, Egham, TW20 0EX UK; 2 Departamento de Estratigrafía y Paleontología, Universidad de Granada, 18002 Granada, Spain

**Keywords:** Palaeontology, Sedimentology

## Abstract

Bioturbation is an important factor for reservoir quality due to the modification of host rock petrophysical properties (i.e., porosity, permeability, and connectivity). However, there is no predictable relationship between bioturbation and its effect on rock properties, due to the variability of the involved ichnological features. A detailed ichnological analysis is necessary to determine how bioturbation affects petrophysical properties in a bioturbated reservoir. Traditionally, ichnological features such as density, tiering, size, orientation, architecture, and fill, have been considered. However, other properties have been undervalued as is the case of lining. Here, we present a detailed study on the effects of *Macaronichnus* burrows, an ichnotaxon usually related to hydrocarbon exploration due to its high concentration in rock notably affecting petrophysical properties. *Macaronichnus*, a subhorizontal cylindrical burrow, is characterized by a well-defined and developed outer rim surrounding the tube core. Our data indicates a clear zonation in porosity according to burrow structure, with the lowest porosity in the tube core and higher values associated with the surrounded rim. This duality is determined by the tracemaker grain selective feeding activity and the consequent concentrated cementation. The organism concentrates the lighter minerals in the tube core fill during feeding, favoring post-depositional cementation during diagenesis and this results in lower porosity than the host rock. However, heavy minerals, mainly glauconite, are located in the rim, showing higher porosity. Our results support the view that ichnological analyses are essential to determine reservoir quality in bioturbated reservoirs, evidencing that other ichnological properties in addition to those traditionally considered must be evaluated.

## Introduction

Bioturbation is an important factor for reservoir quality estimation in the oil and gas industry. Biogenic activities may affect some petrophysical properties such as porosity, permeability, and connectivity^1–3^. This influence on rock properties can be either positive or negative (i.e., either increasing or decreasing porosity, respectively, in comparison to the host rock). Detailed ichnological analyses should be conducted in every bioturbated reservoir to quantify its effect and estimate the reservoir quality^2,4^. Traditionally, attention has been paid to particular characteristics of burrows impacting rock properties and reservoir quality such as density, tiering, size, orientation, architecture, fill, and lining^2,5^. With respect to lining, analyses have focused on its presence or absence^6,7^, and rarely on the petrophysical properties^8,9^. However, thickness and composition of the lining/mantle can be a major feature to be considered for some burrows, as is the case for *Macaronichnus*^10^.

*Macaronichnus* has been commonly associated with reservoir exploitation and then its effect has been analyzed in detail for some deposits^1,9,11–13^. Thus, Gingras et al.^11^ briefly pointed out that porosity distribution in sandstones dominated by *Macaronichnus* is complex and may affect the reservoir quality, but they suggest more research had to be done. Later, Gordon et al.^13^ analyzed the presence of *Macaronichnus* in the Bluesky Formation (Alberta, Canada) to determine that permeability was enhanced in those zones where *Macaronichnus* were abundant compared to similar non-bioturbated intervals. Then, Greene et al.^8^ pointed to differences between lining and filling, and Quaye et al.^9^ found that porosity and permeability in the burrow fill of *Macaronichnus* were higher than in the host sediment in the Funing Formation (Subei basin, China). These studies have shown the effect of *Macaronichnus* and its positive impact in spite of the general idea that bioturbation reduces permeability^4^. Moreover, *Macaronichnus* usually occurs in dense concentrations, commonly occupying more than 70% of the rock^4^, and it just takes very little time for producers (i.e., polychaetes) to do it^14^. At that concentration, it is an important biogenic structure affecting petrophysical properties, fluid flow, and, consequently, oil production. For all these reasons, we decided to analyze the effect of *Macaronichnus* in more detail.

**Figure 1:**
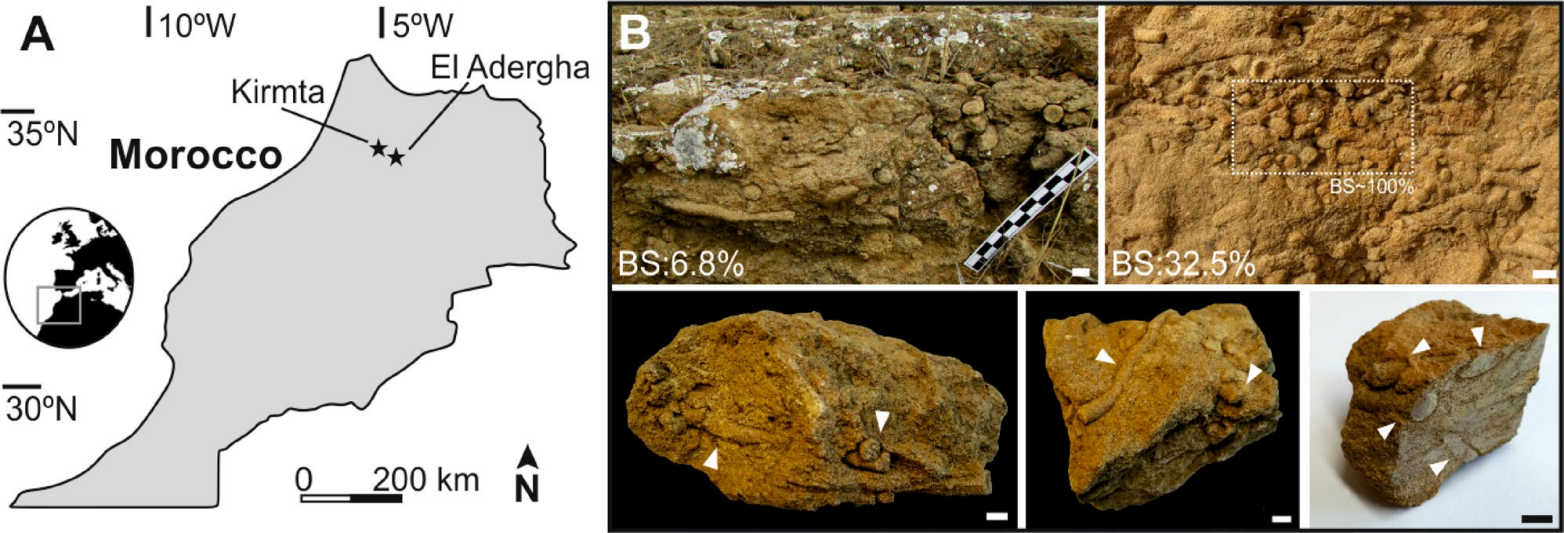
(**A**) Location map of the samples. (**B**) Outcrops and rock samples containing *Macaronichnus* with different bioturbation intensity and overlapping between them. White arrows point to *Macaronichnus* in rock samples. *BS* Bioturbated Surface. Scale bars 1 cm.

*Macaronichnus segregatis*^15^ can be found from Permian to Holocene^2^, mostly in shallow foreshore and shallow subtidal deposits, but it has been also observed in deep-sea sediments such as shelf or slope deposits^16–18^. *Macaronichnus* is usually identified as non-branching, cylindrical, and sub-horizontal burrows from just a few millimeters up to 15 mm diameter, characterized by a clear mineralogical segregation between the cylinder tube core and the surrounding rim^15^. Rim composition shows a concentration of darker and heavy minerals re-sorted by the producer during feeding, while lighter minerals commonly characterize the tube core^2,11^. This rim can be relatively thick, especially in the subichnospecies *Macaronichnus segregatis degiberti*^16,17^. However, the incidence of the rim on petrophysical properties has rarely been considered separately but as part of the entire infilling material (tube core and rim)^9^. Here, we analyze in detail the composition of infilling material from *Macaronichnus segregatis degiberti* differentiating between tube core and rim, focusing on its respective incidence on porosity and then on their particular impact in reservoir quality. This will help us to understand better the relative effect of every single part in porosity, which is essential to provide an accurate estimation of porosity in bioturbated reservoirs. For this study we investigate rock samples including specimens of *Macaronichnus segregatis degiberti* that were collected from two outcrops in northern Morocco: El Adergha (34°4′34.19″N, 4°51ʹ33.43″W) and Kirmta (34°10ʹ15.07″N, 5°14ʹ21.43″W)^18–20^ (Fig. 1A). These outcrops are interpreted as clastic contourite sandstones associated to the paleo-Mediterranean Outflow Water and the Rifian corridor that was connecting the Atlantic Ocean and the Mediterranean Sea during Late Miocene^18–20^. The outcrops belong to the South Rifian corridor (Saiss Basin) and are Late Miocene in age^19–23^. These locations were selected as sampling allows us to evaluate nice examples where *Macaronichnus* specimens are clearly identified with different degrees of bioturbation and overlapping between burrows (Fig. 1B).

## Results

**Figure 2:**
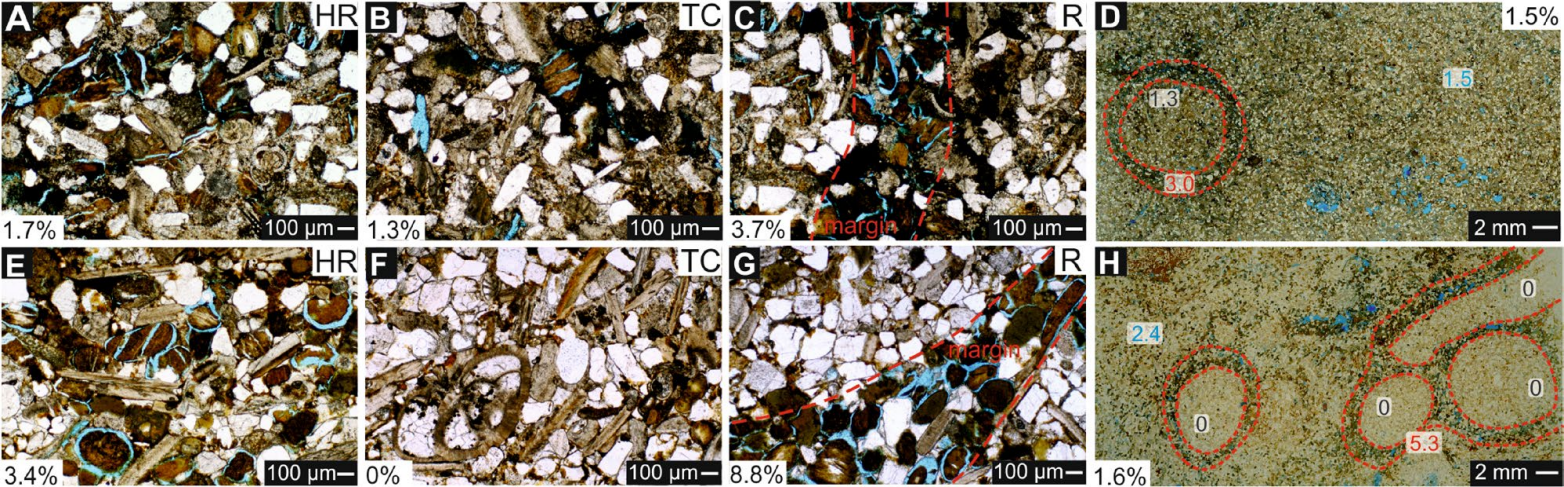
Close-ups from El Adergha (**A**–**D**) and Kirmta (**E**–**H**). (**A**,**E**) Close-ups from host rock (HR). (**B**,**F**) Tube cores (TC). (**C**,**G**) Rims (R). (**D**,**H**) Thin sections. Percentage values show the porosity in every case; in (**C**) and (**G**) this is just referred to the rim. In thin sections (**D**) and (**H**), values measured in tube cores (black), rims (red), and host sediment (blue).

Petrographic analyses reveal that studied clastic contourites, defined as sandstones in the field, have similar proportions of clastic and carbonate components (Table 1). Strictly, they vary between bioclastic limestones and lithic sandstones, both with abundant glauconite. Carbonate clasts consist of bioclasts and some reworked carbonate rock fragments. Bioclasts are mainly bivalve fragments together with other fragments of foraminifera, echinoids, calcareous algae, and rarely bryozoa. Most bioclasts are rounded and clearly reworked. Quartz grains are abundant, feldspar and mica almost absent. Pellets of glauconite are abundant and recognized in all the samples; their distribution is not random and is determined by burrow configuration (Fig. 2, Table 1). Glauconite is mainly concentrated in the rim of *Macaronichnus* burrows; meanwhile the tube core is mostly composed on carbonate components (i.e., carbonate rock fragments and calcite cement) and quartz (Fig. 2, Table 1). Visually, burrow rims are mostly defined by a marked concentration of glauconite pellets. Elongate clasts lie parallel to the margin.

**Table 1:** Mean values of composition and porosity from analyzed samples considering the whole sample (WS), tube core (TC), rim (R) and host rock (HR).

	Quartz	Feld	Mica	Glauc	Carb. R.F	Other R.F	Argill. matrix	Heavy min	Forams	Other biocl	Calcite cement	Dolom	Iron ox./hydrox	Opaq	Por (%)
**El Adergha**															
WS	20.6	0.6	0.0	20.5	15.8	0.6	0.7	0.0	5.0	10.9	21.0	0.2	4.0	0.0	1.5
TC	21.2	0.0	0.0	5.2	22.6	0.0	1.7	0.0	9.7	8.0	28.5	0.0	3.1	0.0	1.3
R	–	–	–	–	–	–	–	–	–	–	–	–	–	–	3.0
HR	19.0	0.7	0.0	18.3	15.8	0.7	1.1	0.0	6.5	9.7	23.7	0.7	3.6	0.4	1.5
**Kirmta**															
WS	15.1	0.0	0.0	22.5	20.6	0.0	1.1	0.0	3.4	15.6	9.6	0.2	11.9	0.2	1.6
TC	23.0	0.0	0.0	2.7	18.7	0.7	0.0	0.0	5.7	12.3	26.0	1.3	9.3	0.3	0.0
R	22.6	0.0	0.0	29.3	6.6	0.0	1.0	Tr.	1.7	18.1	13.2	0.0	7.0	0.3	5.3
HR	17.5	0.0	0.0	18.9	21.6	0.0	0.0	0.0	2.1	13.4	16.5	0.3	9.3	0.3	2.4

*Tr.* Traces, *Feld.* Feldspar, *Glauc.* Glauconite, *Carb.* Carbonates, *R.F.* Rock fragments, *Argill.* Argilliceous, *Biocl.* Bioclasts, *Dolom.* Dolomite, *Opaq.* Opaques, *Por.* Porosity.

When comparing the data of porosity from the whole sample (matrix and burrows) and the host rock (i.e., the matrix), which represent the primary porosity (Table 1), values are slightly affected (2.4% decreases to 1.6% in Fig. 2H) or even remain unaltered (1.5% in El Adergha; Fig. 2D). However, porosity is not homogeneously distributed and it is clearly determined by zonation of the burrows. Tube cores show lower values than host sediment in El Adergha (1.3% vs 3.0%), and they are being even completely non-porous in the case of Kirmta (0.0% vs 5.3%). The highest porosity is located in rims, doubling values recorded in the host sediment in both cases and showing up to 8.8% porosity in some areas (Table 1, Fig. 2).

## Discussion

It is well established that bioturbation may affect reservoir quality^1–3,11,12,21^. Previous studies have demonstrated that *Macaronichnus* is one of the trace fossils that can play an important role in bioturbated reservoirs^9,11,13^, generally having a positive impact. However, the previous studies mostly compared petrophysical properties from the host sediment and the entire burrow, without distinguishing between burrow tube core and outer rim. Our results demonstrate that the presence of *Macaronichnus segregatis degiberti* have, in general, a slightly negative impact on porosity, reducing values less than 1%. However, going deeper, our data show a relevant fact, that is the marked difference between tube core and rim, not only in terms of composition, as is well-known, but also in porosity.

Regarding composition, the tube core is filled with light minerals that are highly cemented, and dark minerals, mainly glauconite, are concentrated in the rims. This compositional zonation is a diagnostic criterion for *Macaronichnus*^15^ and is determined by trace-makers during selective feeding activity^11,15,17^. In regards to porosity, a clear zonation is revealed. Mean porosity in the rims is two times higher than in the host sediment, reaching up to 8.8% porosity in some areas of the rock samples; however, porosity is very low in tube cores. This porosity zonation could be caused by original mineralogical heterogeneity combined with the associated post-depositional cementation^1^. During producer feeding activity, light minerals (mostly quartz and carbonates) are concentrated in the tube core and darker minerals (e.g., glauconite) are placed on the surrounded rim. This zonation causes the concentration of depositional cementation in the tube core. This concentrated cementation produced during diagenesis, generates a porosity zonation, which plays a determinant role in the effect on petrophysical properties. Then, tube cores are characterized by lower porosity values than the surrounded rims.

**Figure 3:**
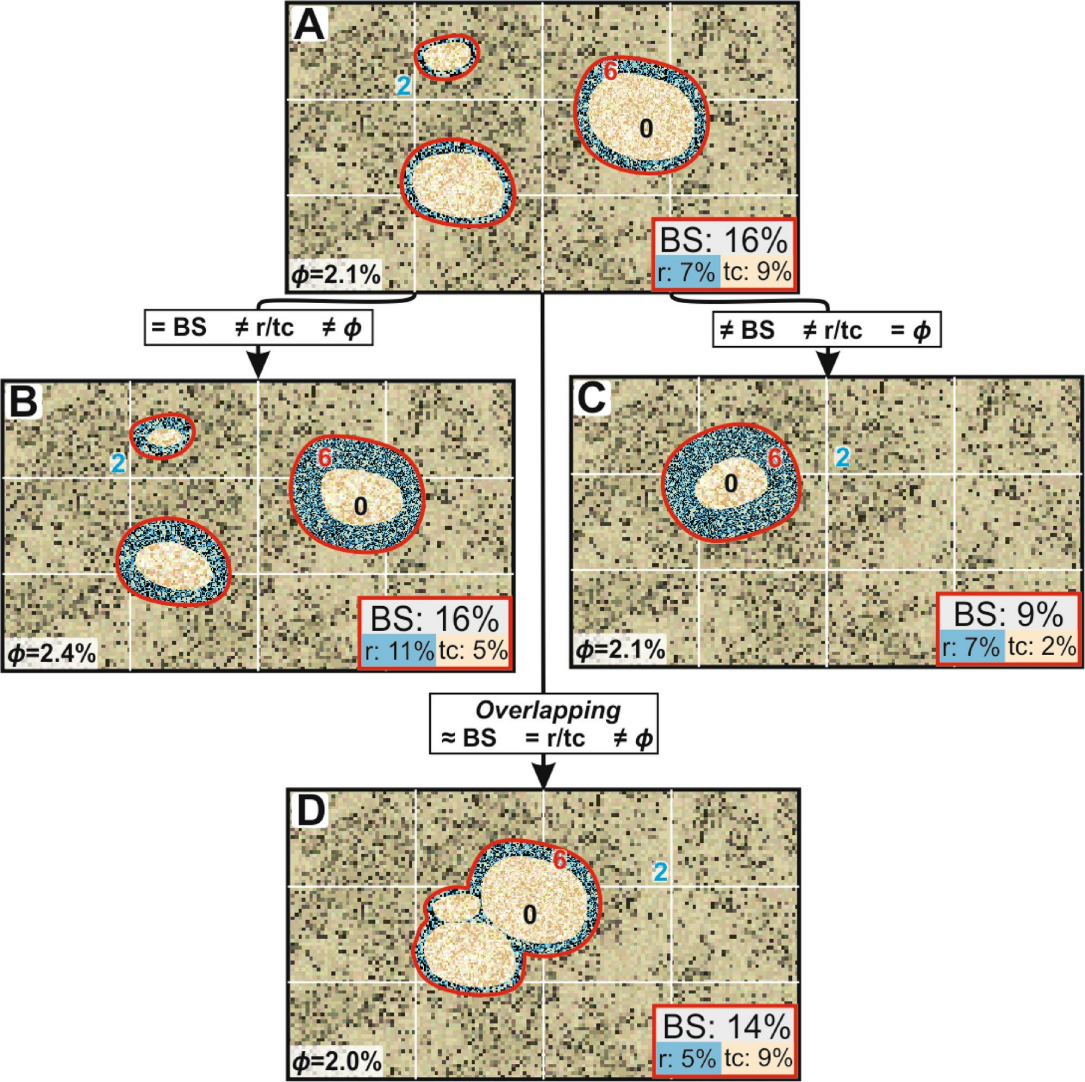
Theoretically modeled porosity effect by *Macaronichnus*. (**A**) Model scenario to be compared with 16% of bioturbated surface (BS) and porosity (*ϕ*) 2.1%. (**B**) Situation with same BS, but different rim/tube core ratio (r/tc), causing an increase in *ϕ*. (**C**) Scenario with same *ϕ* (2.1%) in spite of the lower BS, due to a higher r/tc. (**D**) Overlapped burrows with a very similar BS, but a decrease in rim surface due to crosscutting, reducing *ϕ*. Bold numbers represent porosity from host sediment (blue), rim (red), and tube core (black).

Gingras et al.^11^ reported that porosity distribution in *Macaronichnus* bioturbated sandstone is complex and acts like a dual porosity–permeability system. That means there would be a preferential flow through the burrows and a secondary flow from surrounding sediment into the burrows. Dual permeability systems are found in reservoirs where permeability contrast between matrix and burrows is high, around three orders of magnitude^4^. However, when permeability contrast is lower, a dual-porosity system is developed and there is not a preferential flow through the trace fossils^4^. Based on our results, this can be more complex for *Macaronichnus* as there is not a single flow system for the whole burrow due to the observed zonation. Petrophysical properties from tube core and rim are completely different and then both parts of the burrow should be considered separately when evaluating the effect on reservoir quality. Then, fluids would preferentially flow through the rim and, to identify the flow system, permeability contrast between host sediment and rim has to be evaluated. *Macaronichnus* burrow diameters have been reported from just a few millimeters^11,14,22^ to larger sizes up to 15 mm diameter^16–18,23,24^. However, most of the previous studies do not provide rim thick measurements. Savrda and Uddin^10^ recorded burrow tube cores and rim measurements and noted that tube core diameters ranged from 4 to 19 mm in large specimens. They reported that the rim thickness was variable along the burrow, ranging from about 1 to 5 mm, being thicker at the bottom part of the burrow. This reveals that the rim occupies 25–30% of the volume of the burrows^10^. This fact can also be observed in some of the illustrated figures from previous papers. For example, Rodríguez-Tovar and Aguirre^16^ show large specimens of *Macaronichnus segregatis degiberti* with ~ 15 mm diameter where rim was around 2–3 mm thickness, and Nara and Seike^17^ illustrated that the rim can be even thicker representing half the volume of the actual burrow (Fig. 3 from Nara and Seike^17^).

Therefore, rim thickness must not be underestimated, revealing as a key point to control *Macaronichnus* porosity effect. Especially considering that rim porosity can be two or three times higher than host sediment and represent an important volume portion of the whole burrow (up to 50%). Assuming that *Macaronichnus* are frequently in dense concentrations, commonly representing more than 70% of bioturbation^4^, we can estimate that in some cases burrow rims can represent 35% of the total volume in a bioturbated sample.

Accordingly, to conduct a proper reservoir quality estimation in *Macaronichnus*-bearing rocks, porosities from both zones (rim and tube core) and their relative volumes need to be considered. Another aspect that needs to be considered is the frequent burrow crosscutting between specimens. Several theoretical situations involving different variables such as rim/tube core volume ratios, the degree of bioturbation and the overlapping between burrows are illustrated in Fig. 3, assuming porosity values of 2.0%, 0.0% and 6.0% in the host rock, rim, and tube core, respectively. We established a model scenario considering an example with 16% of bioturbated surface (BS) by isolated burrows, with a rim/tube core (r/tc) volume ratio of 0.77 and porosity (ϕ) of 2.1% of (Fig. 3A). We considered another scenario where burrows occupy the same surface but with thicker rim (r/tc = 2.2), which resulted in higher porosity (ϕ = 2.4%) (Fig. 3B). Whereas in a less bioturbated example (Fig. 3C), we obtained the same porosity value compared to the model scenario (ϕ = 2.1%) due to a higher r/tc volume ratio (r/tc = 3.5). We simulated a final scenario with overlapped burrows (Fig. 3D) where the crosscutting of burrows resulted in a slightly lower surfaces value and r/tc ratio which caused a small reduction of the porosity value compared to the model scenario (ϕ = 2.0%). These four scenarios show how the relative r/tc volume ratios and overlapping of burrows can affect the resulting porosity values. Although the results from these scenarios did not show significant changes in porosity values, but considering that *Macaronichnus* is commonly found representing more than 70% of rock volumes^4^, where rims could represent up to 50% of the burrows^17^ and overlapped, they could significantly modify the resulting porosity values of the host rock.

## Conclusions

*Macaronichnus* has been shown to be a relevant trace fossil for reservoir quality in bioturbated deposits, mainly related to its occurrences as dense concentrations. This study has demonstrated that the porosity effect by *Macaronichnus* is not homogenous in the entire burrow and its distribution is clearly associated with the trace fossil structure. Porosity in the burrow tube core is different to that from the outer rim.

Producers of *Macaronichnus* generate a grain resorting during feeding when they concentrate carbonate components and quartz grains in the tube core fill, and heavy and darker minerals, mainly glauconite, in the rim. Regarding reservoir quality, results reveal that *Macaronichnus* is a complex case and has a dual behavior. Burrow rims have increased porosity with respect to the host sediment and provide positive effects. Fillings in tube cores have lower porosity and provide negative effects.

On this basis, the relative volume occupied by rims and tube cores should be determined to estimate the real effect of *Macaronichnus* on porosity. Any evaluation of the impact of *Macaronichnus* on petrophysical properties, and then on reservoir exploitation, based on just the intensity of bioturbation or the study of the whole burrow must induce significant misinterpretations of relevant scientific and economic implications. This complex dual behavior has been observed for the first time in *Macaronichnus*, but we could not discard that this could also be identified in some other trace fossils with marked internal zonation.

## Materials and methods

Twenty-four rock samples of contourite sandstones including specimens of *Macaronichnus segregatis degiberti* were collected from two outcrops in northern Morocco: El Adergha (34°4ʹ34.19″N, 4°51ʹ33.43″W) and Kirmta (34°10ʹ15.07″N, 5°14ʹ21.43″W)^18–20^, (Fig. 1A). Ten samples were selected encompassing different degrees of bioturbation and variable overlapping between burrows (Fig. 1B). From the 10 samples, 30 thin sections containing *Macaronichnus* were prepared in the Department of Earth Sciences at Royal Holloway University of London and impregnated with blue dyed resin to highlight porosity. Struers EpoFix epoxy mixed with Sudan Blue II powder was used for the resin. Composition of all thin sections was described and eight of the thin sections, where *Macaronichnus* was better defined, were selected to conduct modal analyses. Host rock, tube core, and rim were differentiated for these analyses. Eleven quantitative modal analyses were conducted by determining the composition at 300 points on each thin section using a stepping stage and associated PETROG (Conwy Valley Systems Limited, UK), a software commonly used in quantitative petrography^25,26^, considering the largest intact rectangular area avoiding any marginal alteration. Mineral compositional analyses of tube cores and rims were undertaken by defining an elliptical area within these burrow parts. Rim modal analyses were not possible in samples from El Adergha due to rim size limitations. Porosity was also measured during modal analyses in host rock, tube core, and rim. Additionally, porosity from all the thin sections was also obtained by blue pixels counting using Photoshop^27^, obtaining a representative mean value for the whole samples and from every single part (i.e., host rock, tube core, and rim). Blue pixels were quantified using Color Range Selection Method^28^. This method allows selecting all the blue pixels from an area of interest by clicking on some blue pixels and extending the selection to all the pixels with similar values. Once all the blue pixels are quantified, porosity can be calculated considering the number of pixels composing the area of interest. Intensity of bioturbation was quantified considering the bioturbated surface (BS), using Ichnological Digital Images Analysis Package (IDIAP) quantitative method^28^.

Theoretical scenarios were simulated considering different rim/tube core ratios, degree of bioturbation and overlapping between burrows (Fig. 3). For that, we selected some reasonable porosity values (2.0% for host rock, 0.0% for rims and 6.0% for tube core burrows) based on thin sections observations. Resulting porosity was calculated using the selected porosity values for every zone considering the relative surface of every single part in every scenario.

## Data Availability

All data analysed during the present study are summarized in this published article. Analyzed thin sections are available under request.
